# Mitochondrial Management of Reactive Oxygen Species

**DOI:** 10.3390/antiox10111824

**Published:** 2021-11-17

**Authors:** Gaetana Napolitano, Gianluca Fasciolo, Paola Venditti

**Affiliations:** 1 Dipartimento di Scienze e Tecnologie, Università degli Studi di Napoli Parthenope, Via Acton, 80133 Naples, Italy; gaetana.napolitano@uniparthenope.it; 2 Dipartimento di Biologia, Università di Napoli Federico II, Complesso Universitario Monte Sant’Angelo, Via Cinthia, 80126 Naples, Italy; gianluca.fasciolo@unina.it

**Keywords:** oxygen consumption, OxPhos, ROS generation, ROS removal, enzymatic antioxidants, low-molecular-weight antioxidants

## Abstract

Mitochondria in aerobic eukaryotic cells are both the site of energy production and the formation of harmful species, such as radicals and other reactive oxygen species, known as ROS. They contain an efficient antioxidant system, including low-molecular-mass molecules and enzymes that specialize in removing various types of ROS or repairing the oxidative damage of biological molecules. Under normal conditions, ROS production is low, and mitochondria, which are their primary target, are slightly damaged in a similar way to other cellular compartments, since the ROS released by the mitochondria into the cytosol are negligible. As the mitochondrial generation of ROS increases, they can deactivate components of the respiratory chain and enzymes of the Krebs cycle, and mitochondria release a high amount of ROS that damage cellular structures. More recently, the feature of the mitochondrial antioxidant system, which does not specifically deal with intramitochondrial ROS, was discovered. Indeed, the mitochondrial antioxidant system detoxifies exogenous ROS species at the expense of reducing the equivalents generated in mitochondria. Thus, mitochondria are also a sink of ROS. These observations highlight the importance of the mitochondrial antioxidant system, which should be considered in our understanding of ROS-regulated processes. These processes include cell signaling and the progression of metabolic and neurodegenerative disease.

## 1. Introduction

Mitochondria are double-membrane organelles located in the cells of most eukaryotic organisms, where they perform a wide range of important functions. They contribute to the regulation of intracellular calcium concentration and control the oxidation of fatty acids, as well as being involved in the metabolism of steroids and some amino acids, and the synthesis of urea and phospholipids. However, their main function is to provide most of the energy required for cellular endergonic reactions, such as the synthesis of adenosine 5′-triphosphate (ATP) by the electron transport chain (ETC) and oxidative phosphorylation. This explains why a disturbance of mitochondrial function, due to damage of the mitochondrial components, can cause an impairment of cellular function and even cell death.

The electron flux along the mitochondrial respiratory chain also determines the production of oxygen radicals and other reactive species, referred to as reactive oxygen species (ROS) [1]. ROS include species that are highly reactive, such as the hydroxyl radical (^•^OH), and species with a low reactivity, such as the superoxide (O_2_^•−^) and hydrogen peroxide (H_2_O_2_). ROS can oxidize and damage cellular components, including mitochondria, altering their functionality.

Mitochondria produce ROS at a rate that depends on cellular pathophysiological conditions and is low under normal conditions. However, mitochondrial antioxidant systems, composed of enzymatic and non-enzymatic antioxidants [2], largely remove ROS produced by mitochondria.

Under conditions where ROS production increases beyond mitochondrial antioxidant capacity, some components of the mitochondrial electronic chain, or Krebs cycle enzymes, may be deactivated. In these conditions, ROS reaching the cytosol also increase, and can be neutralized here by the antioxidant system of the cell or can damage the cellular components [3].

Several sources of ROS are present in the cell, but mitochondria are considered to be their main source [4].

Extensive experimental evidence indicates that the systems that evolved to protect mitochondria against endogenously produced ROS can also scavenge ROS produced from other cellular sources. [5]. This observation indicates that mitochondria can act as an intracellular sink for ROS, which contrasts with the usually recognized role of the organelles as ROS producers.

However, under which conditions and which part of the mitochondrial population performs a sink function is not yet established. In this review, we analyze the role of mitochondria as an energy source and in ROS generation. We then describe the mitochondrial systems involved in ROS removal. Finally, we analyze the current knowledge on how the mitochondrial antioxidant system can remove ROS produced by other cellular sources.

## 2. Mitochondria as Source of Metabolic Energy Production

### 2.1. Mitochondria and ATP Synthesis

**Figure 1:**
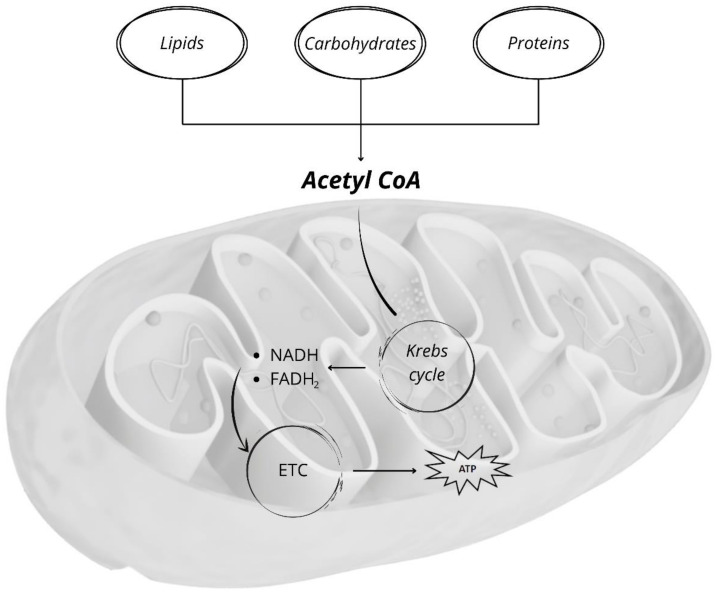
Schematic representation of the mitochondrial involvement in the catabolism of lipids carbohydrates and proteins and ATP production. ETC: electron transport chain. ATP: adenosine triphosphate.

The Krebs cycle plays a crucial role in the energy transduction process that leads to the generation of ATP (Figure 1). The acetyl groups resulting from the catabolism of lipids, carbohydrates and proteins converge in acetyl-coenzyme A. The Krebs cycle leads to the oxidation of the carbon atoms of the acetyl-coenzyme A acetyl groups, converting them into CO_2_ and transferring electrons to the nicotinamide adenine dinucleotide (NAD^+^) and flavin adenine dinucleotide (FAD). The reduced forms of these molecules (NADH and FADH_2_) are reoxidized by the transfer of electrons to the mitochondrial complexes of the mitochondrial electron chain and, eventually, to oxygen in a multi-stage process, in which the energy, resulting from the decrease in the potential of electrons, is gradually released.

Complex I (NADH-ubiquinone oxidoreductase) consists of 46 different subunits and including a flavoprotein and six iron-sulfur centers [6]. It is L-shaped, the long arm is an integral membrane protein, and the short arm protrudes into the matrix and contains the active centers FMN and NADH [7].

Complex II (succinate-ubiquinone reductase) is a component of both the Krebs cycle and the mitochondrial respiratory chain. It is an integral protein with covalently linked FAD and iron-sulfur centers in the extrinsic domain of the membrane [8].

Complex III (ubiquinol-cytochrome c oxidoreductase) is a dimer with each monomer, consisting of 11 subunits. The redox centers, the hemes b562, b566 and c1 and the cluster [2Fe-2S], are associated with 3 of the 11 subunits of each monomer [9].

The final catalyst of the electron chain is the complex IV, the cytochrome c-O_2_ oxidoreductase (COX). COX is a large integral membrane protein which contains two hemes, cytochrome a and cytochrome a3, and two copper centers, the CuA and CuB centers [10].

Parts of the mitochondrial respiratory chain complexes form an ordered structure called “supercomplex” [11], which plays important role in efficient energy production [12], the stabilization of complex I [13], and the prevention of ROS generation [14].

Electrons transfer from NADH and succinate to Complex I and Complex II, respectively. Subsequently, complexes I and II transfer electrons to ubiquinone. This latter is a fat-soluble benzoquinone, which possesses a long isoprenoid side chain that is laterally diffusible in the phospholipid layers of the inner membrane. Electrons pass from ubiquinone through Complex III to cytochrome c (Cytc). Cytochrome c is another mobile vector weakly connected to the outer surface of the inner mitochondrial membrane by electrostatic interactions; it interacts with the cytochrome c1 of Complex III and accepts electrons [15].

Reduced cytochrome c moves along the membrane surface and interacts with Complex IV subunit II by electrostatic interactions, simultaneously transmitting electrons from Complex III to CuA in Complex IV. The latter removes an electron from four reduced cytochrome c molecules (Fe^2 +^ -heme) by oxidizing them to Fe^3+^-cytochrome c. It then transfers the four electrons to the oxygen, which is the final acceptor of the electrons. Therefore, oxygen forms water by a tetravalent reduction.

The flow of electrons from the electron donor, NADH or succinate, to the acceptor, O_2_, occurs due to the oxidation potential of the components in the electron transfer chain. Electrons move to compounds with more positive oxidation potentials. The chemical energy released by the decrease in the redox potential of electrons passing through the respiratory complexes serves to pump protons from the mitochondrial matrix to the intermembrane space. In this way, a proton motor force consisting of an electric (ΔΨm) and proton (ΔpH) gradient [16] is created.

The process by which this force brings protons back into the matrix via the fifth mitochondrial complex (Complex V, ATP synthase), resulting in the synthesis of ATP, is called oxidative phosphorylation (OxPhos) [17].

The ADP/ATP translocase or adenine nucleotide translocator (ANT) exports the ATP to the cytosol to do useful “work” in the cell [18].

### 2.2. Regulation of OxPhos Activity

**Figure 2:**
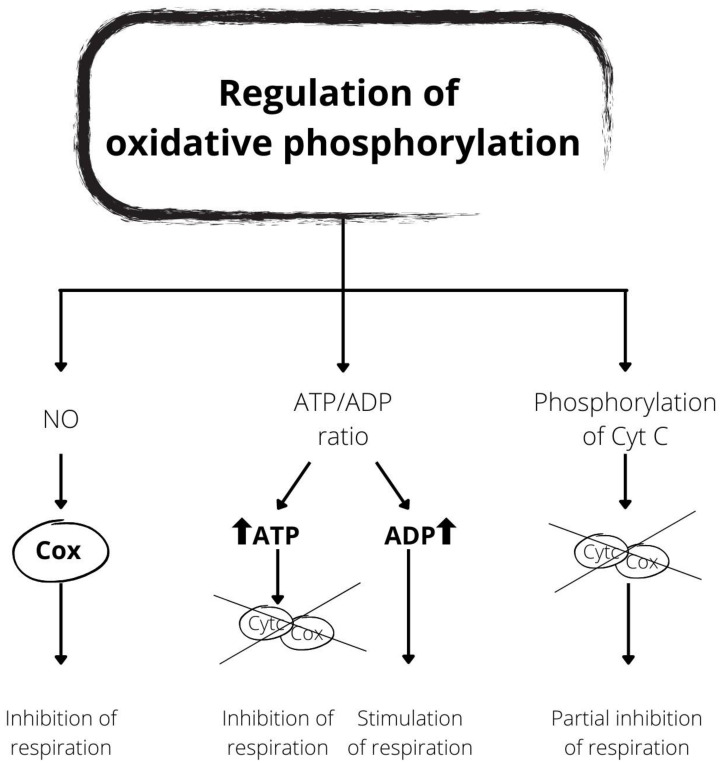
Schematic overview of the main factors involved in OXPHOS regulation. NO, nitric oxide; Cox, cytochrome oxidase; Cytc, cytochrome C. ATP: Adenosine triphosphate. ADP: Adenosine diphosphate.

Due to its essential role, OxPhos is finely tuned for the optimal functioning of an organism (Figure 2). Cytc and COX are the respiratory components primarily involved in regulation. Indeed, in intact mammalian cells, the electron transfer reaction from Cytc to oxygen through COX is the limiting phase of the electron transport chain under physiological conditions [19,20]. This step is also tightly regulated. Indeed, several regulatory mechanisms, including the allosteric regulation of COX and Cytc (e.g., by the ATP/ADP ratio) and reversible post-translational changes, including phosphorylation, were demonstrated [21,22].

Three metabolites, O_2_, NO, and ADP, appear as the main regulators of OxPhos function.

In blood vessels, NO produces vasodilation and increases blood flow and the release of O_2_ to tissues [23]. In mitochondria, NO inhibits respiration through a rapid and reversible inhibition of COX [24]. NO binds to the O_2_ site of the a3 domain of the heme of cytochrome oxidase in the reduced state, competing with O_2_ [25]. Therefore, NO significantly increases the O_2_ concentration at which the O_2_ absorption rate is semi-maximum [26]. This effect allows for the redistribution of oxygen in sites where the O_2_ concentration is low and can be a mechanism for preserving areas with critical oxygen levels [27].

The energy requirements, expressed as cytosolic ADP concentration or phosphorylation potential ([ATP]/[ADP] [Pi]), determine the cellular rates of respiration and ATP synthesis. As cellular energy demand increases, the breakdown of ATP into ADP and Pi increases, and the phosphorylation potential decreases. With more ADP available, the rate of respiration increases, causing ATP to regenerate.

The dependence of the respiratory rate on the concentration of ADP is appointed as a respiratory control or acceptor control. Mitochondrial respiration in the presence of a respiratory substrate but without ADP is called “controlled respiration” or “at rest” (State 4). The respiration with both the respiratory substrate and ADP is called “active respiration” (State 3) [28]. The rate of the respiration of the mitochondria isolated from several tissues is 5 to 8 times higher in State 3 than in State 4. Therefore, the respiratory control ratio (RCR) assumes a value between 5 and 8.

ATP acts as a negative feedback inhibitor of the interaction between Cytc and COX through binding to both proteins, and thus regulating the speed of the electron flux [29]. The allosteric inhibition exhibited by ATP uncompetitively acts by transforming the high-affinity Cytc–COX binding site into a low-affinity site [30]. Arg91 is the amino acid of Cytc involved in ATP binding. When ATP occupies this site, the electron transfer activity of Cytc significantly reduces [31]. This effect is likely due to structural changes in both proteins and electrostatic changes when ATP, negatively charged, binds to Cytc and COX. Therefore, the activity of the electron transport chain is adapted to the energy demand through the ATP/ADP ratio as an intrinsic measure of the energy state of the cell.

Another regulatory mechanism, mediated by phosphorylation, was discovered in Cytc, isolated from the cardiac [32] and liver [33] tissues of cows, under conditions in which the state of in vivo physiological phosphorylation was preserved. Cytc tyrosine phosphorylation was found in both tissues, but cardiac Cytc was phosphorylated on Tyr97 [32], while hepatic Cytc was phosphorylated on Tyr48 [33]. The phosphorylation of both tyrosine residues resulted in the partial inhibition of the reaction with the isolated COX leading to the control of respiration. Since then, it was corroborated that the phosphorylation of Cytc occurred in a highly tissue-specific way [21]. Thr28 phosphorylation of Cytc was mapped in the bovine kidney [34], and a second phosphorylation site, Thr58, was subsequently identified in the rat kidney [35]. Ser47 was mapped in rat and pig brains under basal conditions [36], while Tyr97 was identified in post-insulin treatment in rat and pig brains [37]. These phosphorylations of Cytc also lead to functional changes, including altered reaction kinetics with COX.

Cytc is a small protein, and this could explain why its modifications, even if not directly part of the COX interaction site, according to crystallographic data, can affect or interfere with the optimal Cytc–COX binding. For example, the Cytc and COX interaction is mainly mediated by electrostatic interactions of positively charged lysine residues on Cytc and negatively charged COX residues, in addition to hydrophobic interactions across the binding interface [38]. Therefore, depending on their specific position on Cytc, the introduction of negative charges after phosphorylation can affect and interfere with the optimal binding of Cytc to COX and reduce the reaction rate.

## 3. Mitochondria as Sources of ROS

More than 50 years have passed since the mitochondrial generation of H_2_O_2_ in the presence of respiratory substrates was discovered [39]. A few years later, it was discovered that mitochondria contain superoxide dismutase (SOD) [40] and produce O_2_^−•^ [41]. These findings suggested that mitochondria produce H_2_O_2_ by the dismutation of O_2_^−•^.

In mammalian mitochondria, the genesis of most of the produced ROS involves the same electron transfer pathways implicated in the oxidation of nutrients and ATP biosynthesis. In fact, under physiological conditions, a small part of the electrons in the electron transport chain does not follow the usual transfer order but exits directly from the ETC and interacts with O_2_ to produce O_2_^−•^ through a univalent reduction.

### 3.1. Sites of ROS Production

The ROS produced during the tetravalent reduction of O_2_ by cytochrome oxidase are not released into the surrounding medium. Conversely, the superoxide generated by the auto-oxidation of carriers of other respiratory complexes is released into the surrounding medium. For a long time, evidence was provided that secondary O_2_ reaction sites localized in Complex I [42,43] and Complex III [44]. Only several years later, it was reported that potential ROS production sites localize in mitochondrial Complex II [45,46] and in various mitochondrial enzymatic components, as well as components of the respiratory chain [47,48]. However, the exact sites of ROS generation and their relative contribution in vivo remains an unsolved problem.

To date, eleven different sites involved in oxidation and electron transport have been identified as ROS producers in mammalian mitochondria [49]. These sites give electrons to oxygen to produce superoxide or hydrogen peroxide. However, it is not well established if a site transfers one electron to oxygen to generate a superoxide radical or two electrons to generate hydrogen peroxide, or both of these.

The electron flow from metabolites to O_2_ occurs because of the oxidation potential of the mitochondrial components. The electrons flow into the NADH/NAD^+^ pool via the NAD-bound dehydrogenases. The 2-oxoacid dehydrogenase complexes catalyze the oxidative decarboxylation of several 2-oxoacids to acyl-CoA and NADH. The 2-oxoacid dehydrogenase complexes comprise: (1) 2-oxoglutarate dehydrogenase (OF site) [50], (2) pyruvate dehydrogenase (PF site) [50], (3) branched-chain 2-oxoacid dehydrogenase (site BF) [50], and (4) aminoadipate dehydrogenase (site AF) [51]. The dihydrolipoamide dehydrogenase of each complex contains a FAD, a powerful electron leak site, which can generate superoxide/hydrogen peroxide. The 2-oxoacid dehydrogenase complexes exclusively generate superoxide and/or hydrogen peroxide in the matrix space being localized in the mitochondrial matrix or loosely attached to the inner face of the inner membrane.

The electrons pass from the NADH to the flavin-containing site (site IF) of Complex I and then through the quinone-binding site (site I_Q_) to the ubiquinone-bound dehydrogenase pool. The I_F_ site generates superoxide [52,53] exclusively into the matrix space [52]. The localization of the I_F_ site near the tip of the hydrophilic arm of complex I [54], which protrudes into the mitochondrial matrix, accounts for superoxide release into the matrix.

The I_Q_ site generates most of the superoxide/hydrogen peroxide when succinate or glycerol 3-phosphate drive oxygen consumption. In this case, the generation of superoxide/hydrogen peroxide depends on the reverse transport of electrons. In this phenomenon, the high ubiquinol/ubiquinone ratio (QH_2_/Q), and the high protonmotive force, due to the electron transfer through complexes III and IV, push electrons into Complex I against the redox potential [55].

Ubiquinone-bound dehydrogenases transfer electrons to the pool of QH_2_/Q. These dehydrogenases are (1) the site G_Q_ (mitochondrial glycerol-3-phosphate dehydrogenase), (2) the site E_F_ (electron-transfer flavoprotein (ETF): Q oxidoreductase system), (3) the site D_F_ (dihydroorotate dehydrogenase), and (4) the site II_F_ (complex II).

Site II_F_ produces a negligible amount of ROS in normal conditions. This amount increases in disease related to the Complex II mutation mainly due to the site IIF [56]. Site II_F_ releases ROS exclusively in the matrix because the flavoprotein localizes on the matrix side of the inner mitochondrial membrane [50].

From QH_2_, the electrons transfer to the outer Q-binding site of complex III (site III_Qo_). After that, they pass to the center III_Qi_ in the Q-cycle through cytochrome b566 and to oxygen through the cytochrome c and complex IV.

The III_Qo_ site [57] generates superoxide. It locates on the outer side of complex III [58], and releases superoxide both in the matrix and in the intermembrane space [58].

### 3.2. Regulation of ROS Production

The reaction of superoxide formation is of second order since its rate (d[O_2_^•−^]/dt) depends on the product of two factors: the local concentration of O_2_ and the concentration of the electron carriers capable of transferring an electron at O_2_, generating O_2_^•−^; in reduced form, (R^•^) (autoxidizable carrier). The latter, for its part, depends on the concentration of the electron carriers (C) and their fraction in the reduced form (F):

$$d[O_{2}{}^{•-}]/dt=k\cdot [O_{2}]\cdot [R^{•}]$$

Another factor affecting the reaction rate is the rate constant (k) for the reaction between electron carrier and O_2_, which has the dimensions of the inverse of the product of concentration and time.

The concentration of mitochondrial carriers responsible for O_2_^•−^ production varies according to animal species, tissue, age, and hormonal state [59]. This may underlie many of the differences in maximum ROS production rates between tissues. Thus, a high concentration of complex I in rat heart mitochondria compared to pigeon heart mitochondria explains why rat mitochondria releases significantly more H_2_O_2_ than pigeon mitochondria [60].

Most likely, the main factor influencing of O_2_^•−^ production rate is the fraction of the autoxidizable carrier in the reduced form. The value of this fraction strongly depends on the rate of electron flow. Indeed, when this rate increases, F decreases and the rate of O_2_^•−^ also decreases. This inverse dependence of (R^•^) on the rate of electron flow explains the observation that the rate of O_2_^•−^ production during State 4 is higher than during State 3. In the presence of ADP (State 3), the electrochemical gradient on both sides of the mitochondrial inner membrane is dissipated through the ATP synthetase complex leading to ATP synthesis. The rate of electron flow is high, whereas both the F value and rate of O_2_^•−^ are low. In the absence of ADP (State 4), the flux of H^+^ through the ATP synthetase stops. In this condition, the gradient of H^+^ increases, the electron flow slows down and the reduction degree of the respiratory chain and the generation of superoxide increases.

A lowering of electrochemical gradient can also be obtained by inducing a small proton leak through the inner mitochondrial membrane, which stimulates oxygen consumption and, in parallel, decreases the reduction degree of mitochondrial, autoxidizable carriers, and thus lowering the ROS production. In in vitro experiments, this phenomenon can be triggered by substances (uncouplers) that allow a rapid diffusion of protons in the mitochondrial matrix, thus causing an acceleration of the electronic flow.

The regulated, moderate (“mild”) uncoupling of mitochondrial oxidative phosphorylation was suggested as a feasible therapeutic strategy for the regulation of the intracellular and intramitochondrial ROS level [61].

Fatty acids are mild natural uncouplers [62]. In their protonated form, they can cross the mitochondrial inner membrane followed by deprotonation in the matrix side. The anionic form of the fatty acid completes the cycle by returning to the cytosolic side.

Mitochondrial uncoupling proteins (UCPs) were considered as potential mild uncouplers. The relationship between ROS production and UCPs activity was revealed in 1997 in experiments where GDP, an inhibitor of UCP1, caused an increase in ΔΨ and ROS production [63].

Different from the uncouplers, the presence in in vitro experiments of inhibitors of the respiratory chain, such as rotenone (an inhibitor of Complex I), or antimycin (an inhibitor of Complex III) causes the carriers upstream from the site of inhibition to become fully reduced and increases the formation of O_2_^•−^.

It has long been known that tissue oxygen tension is much less than that in ambient air, and that the oxygen gradient can be readily observed at the intracellular level [64].

Because the apparent K_m_ of cytochrome oxidase for O_2_ is very low (1 μM) [65], at low intracellular levels of oxygen, the amount of oxygen is still sufficient for saturating cytochrome oxidase and maintaining normal mitochondrial respiration [66]. Conversely, the effect of the oxygen tension on the rate of ROS production is more controversial.

Boveris and Chance [67] first demonstrated that mitochondrial ROS production enhances under hyperoxic conditions. This observation found support in previous studies, which showed that even the generation of O_2_^•−^ or H_2_O_2_ by submitochondrial particles [68] and isolated respiratory complexes [53] increased when the O_2_ concentration increased above the usual atmospheric level of 21% O_2_. This increase is roughly proportional to the concentration of O_2_, at least over the lower range of its supraphysiological concentrations. The marked increase in the formation of O_2_^•−^ or H_2_O_2_ under hyperbaric conditions and its immediate onset, provided support at the molecular level for the early hypothesis that oxygen toxicity is in part a consequence of increased rates of the formation of intracellular highly reactive species [69].

Studies dealing with the effects of the decreased concentration of O_2_ showed that isolated complex I O_2_^•−^ production decreases linearly with the lowered O_2_ concentration [53], and that H_2_O_2_ production by isolated mitochondria decreases when the concentration of O_2_ is lowered below that of an air-saturated medium [70]. However, a subsequent study has not confirmed the above observations. Indeed, it was found that the rate of H_2_O_2_ production was almost unaffected by changes in an oxygen concentration ranging from 250 µM (approximately ambient oxygen) to as low as about 5–7 µM (intracellular range) and only decreased when the O_2_ concentration decreased below 5 μM [71].

It was suggested that the dependence of mitochondrial ROS production on O_2_ concentration may be a relevant factor in changes in ROS production in vivo. This is because the levels of extracellular O_2_ change with the physiological state, and there are O_2_ concentration gradients between the bloodstream and the mitochondria, at which the O_2_-consuming cytochrome oxidase locally lowers O_2_ levels. [72,73]. Therefore, changes in the rate of O_2_ consumption by mitochondria may be an important aspect of modifying O_2_^•−^ production in vivo by altering the local O_2_ concentration [72].

A substance able to modify O_2_ concentration and ROS production is nitric oxide (NO). Physiological levels of NO compete with O_2_ for cytochrome oxidase when O_2_ concentration is low, effectively raising the apparent K_m_ of this enzyme [74] and may, therefore, alter the levels of O_2_ around mitochondria, leading to changes in O_2_^•−^ production [75,76].

The final factor affecting the rate of O_2_^•−^ production by electron carriers is the rate constant of their reaction with O_2_. The reaction between protein-bound electron carriers and O_2_ to form O_2_^•−^ seems to be verified through electron tunnels from the electron donor to O_2_. The reaction rate depends on the distance between O_2_ and the electron donor [77]. This movement is similar to that of electrons along the respiratory chain that occur by electron tunnelling from carrier to carrier, with a maximum distance of about 14 Å between each carrier for effective tunnelling to occur [78]. This distance likely forces the reaction between protein-bound electron carriers of the respiratory chain with O_2_ to form O_2_^•−^, while most of the proteins act as insulators to keep O_2_ at a safe distance from the carriers, and thus minimize O_2_^•−^ production [78]. As a result, O_2_^•−^ production likely occurs at sites where O_2_ can closely approach electron carriers.

The rate of electron transfer from protein-bound electron carriers to O_2_ was studied for flavoenzymes that activate O_2_, where the reduction of O_2_ to O_2_^•−^ is often a precursor to further reactions [79]. For the flavoenzyme glucose oxidase, the rate of reduction of O_2_ to H_2_O_2_ is ∼10^6^ M^−1^ s^−1^ [80], and, since the rate-limiting step is the reduction of O_2_ to O_2_^•−^ [80], this indicates that the production of O_2_^•−^ by electron carriers bound to protein can be rapid. Of course, the production of H_2_O_2_ is a consequence of the normal physiological function of glucose oxidase, and for other flavoproteins, where the electron transfer to O_2_ to form O_2_^•−^ may be a side reaction, the rate varies over five orders of magnitude [81]. Even so, the rate of reduction of O_2_ by the reduced FMN of complex I to form O_2_^•−^ is ∼40 O_2_^•−^ min^−1^ [53], corresponding to a second-order rate constant of ∼10^3^ M^−1^ s^−1^. Thus, the k for O_2_^•−^ production as a side reaction by electron carriers bound to proteins can be rapid, although K is likely to vary greatly with the electron donor’s environment and its accessibility to O_2_. Therefore, protein alterations that allow O_2_ to get closer to the electron carrier, such as damage, mutations, post-translational modifications, conformational changes, or quaternary interactions, could lead to dramatic changes in the rate of O_2_^•−^ production and potentially perform a regulatory function. Such factors determining the k-value for the reaction of O_2_ with protein-bound electron carriers could result in mitochondrial O_2_^•−^ production, but little is known about how k can be varied by different enzymes within mitochondria.

## 4. Mitochondrial Antioxidant System

**Figure 3:**
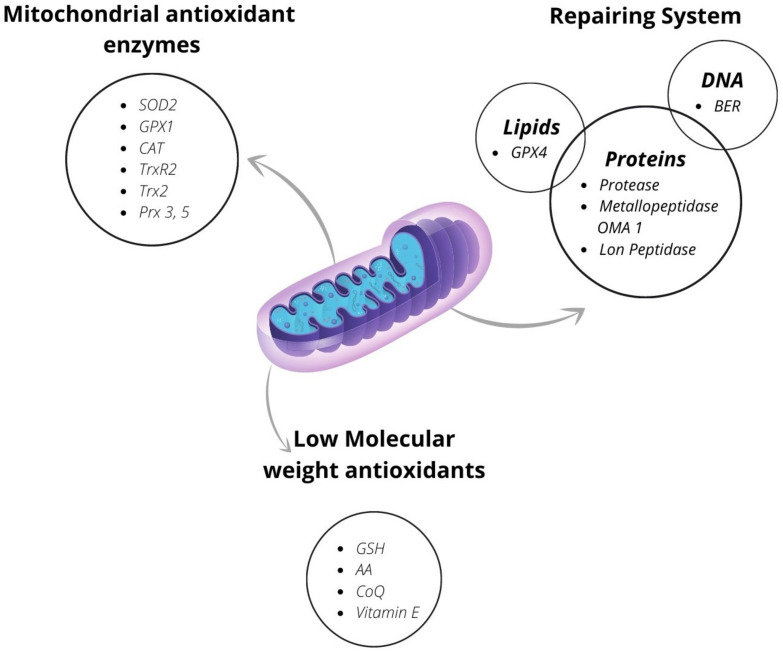
Components of the antioxidant system in the mitochondria SOD2: superoxide dismutase 2; Cat: catalase; GPX1 glutathione peroxidase 1; TrxR2: thioredoxin reductase 2; Trx2: thioredoxin 2; Prx 3, 5: peroxiredoxin 3 and 5; GPX4: glutathione peroxidase 4; BER: base-excision repair; GSH: reduced glutathione; CoQ: coenzyme Q; AA: ascorbic acid.

In mitochondria, the antioxidant defense system articulates on various levels. In some cases, it is possible to find a preventive antioxidant system, but in all mitochondria, there is the interception system and the repair system (Figure 3).

### 4.1. Preventive Antioxidant System

In spermatocytes, neurons, and cardiomyocytes, all tissues with sustained metabolic activity, there is a specific type of ferritin [82] localized in the mitochondria, called mitochondrial ferritin (FtMt), which avidly sequestrates the ferrous ion [83], thus preventing the Fenton reaction-induced ^•^OH generation [83]. FtMt is a key mitochondrial iron storage protein with a high homology to the heavy chain of cytosolic ferritin (FtH) [83,84,85]. FtMt has a ferroxidase activity and catalyzes the conversion of Fe^2+^ to the ferric form for storage in the FtMt spherical shell, which can accommodate up to 4000 iron atoms. It was proposed that the limited expression of FtMt to some tissue depends on its high iron affinity that could lower the iron level in the cytosol by too great an amount [83]. On the other hand, it was shown that in FtMt-overexpressing mice, the regulation of iron metabolism was not significantly modified [86], but that FtMt exerted significant protective effects under pathological conditions, such as in Alzheimer’s disease and Parkinson’s disease.

Wang et al. [87] reported that FtMt levels upregulate in the ischemic brains of mice. Moreover, mice lacking FtMt experience more severe brain damage and neurological deficits, accompanied by the typical molecular features of ferroptosis (increased lipid peroxidation) after cerebral ischemia-reperfusion (I/R), suggesting that FtMt plays a critical role in protecting against cerebral I/R [87].

### 4.2. Mitochondrial Interception System

#### 4.2.1. The mitochondrial Antioxidant Enzymes

Most superoxide or hydrogen peroxide production sites release their product to the mitochondrial matrix, as they are either located in the matrix or located on the inner face of the inner membrane facing the matrix. The superoxide released into the matrix crosses the inner membrane only slowly, so the steady-state level of the matrix superoxide depends on the speed of production at the different sites and the rate of consumption [49].

In the matrix, the enzyme that converts the O_2_^•−^ in H_2_O_2_ is the manganese-dependent superoxide dismutase (MnSOD), also called SOD2 [88]. This enzyme is a tetramer composed of identical subunits each containing one manganese atom. The acetylation state of the two residues of lysine regulates the activity of the enzyme through the control of the superoxide access to the active site of the enzyme [89,90]. The acetylation of one of these lysines inhibits the enzyme and depends on the activity of the mitochondrial electronic chain. Acetylation increases when the mitochondrial chain activity reduces [91,92]. Sirtuin-3, which requires NAD^+^ for its function, operates deacetylation. When ETC activity reduces, NADH accumulates and, consequently, the NAD^+^ level reduces, which in turn reduces Sirtuin-3-mediated SOD2 deacetylation and, therefore, SOD2 activity [93].

It was recently suggested that SOD2 broadcasts the redox signals generated by mitochondria to distant sites in the cytosol, nucleus or even outside the cell [92]. In the presence of SOD2, O_2_^•−^ is converted to H_2_O_2_ at a 2:1 ratio. H_2_O_2_ is a freely diffusible oxidant, and it has a prominent role as a regulator of signaling systems based on redox-sensitive thiol switches.

In the intermembrane space (IMS) of higher eukaryotes there was a minor fraction (less than 5%) of the cytosolic enzyme CuZnSOD (SOD1) [94], which dismutated the released superoxide.

After cytosolic translation, a small amount of SOD1 enters the mitochondria in an unfolded state using the outer mitochondrial membrane translocator, TOM [95]. The post-translational modifications of SOD1 are necessary for the functionality of the enzyme but also affect its subcellular localization. In the IMS, the copper chaperone of SOD (CCS) causes the formation of disulfide bonds and the insertion of copper metal, thus inducing the maturation of SOD1, which remains inside IMS [95].

The SODs-catalyzed reaction of O_2_^•−^ dismutation is in competition with the reaction between O_2_^•−^ and NO^•^, which leads to the formation of peroxynitrite. Peroxynitrite is a powerful biological oxidant involved in various forms of free-radical-induced tissue damage. Thus, the SODs reaction removing O_2_^•−^ in systems containing nitric oxide reduces the formation of the highly reactive peroxynitrite.

The reaction catalyzed by SODs with O_2_^•−^ has a rate constant near to the diffusion control limit (∼1–2 × 10^9^ M^−1^·s^−1^), but the rate constant of the reaction between O_2_^•−^ and NO^•^ is of one order of magnitude higher. On the other hand, the concentration of SOD in mitochondria is high enough (about 10–20 μM in mammalian) to prevent O_2_^•−^ from reacting with NO^•^, whose level under normal conditions is very low (10–100 nM). When NO^•^ levels rise due to the overactivation of nitric oxide synthase, or the induction of inducible nitric oxide synthase (iNOS), more peroxynitrite is generated [96] (Radi, 2018). Mitochondrial nitrosative damage is exacerbated by the inactivation of MnSOD due to peroxynitrite-induced nitration [96].

The importance of SOD2 for mitochondria was also established through the studies on mice deficient in Sod2. Homozygous mutant Sod2 mice displayed neonatal lethality due to the superoxide-induced inactivation of iron-sulfur centers in OXPHOS and citric acid cycle enzymes [97,98]. In contrast, the heterozygous mutant Sod2 animals have a partial OXPHOS defect involving a reduced respiratory control ratio (RCR) and an increased propensity for the opening of the mitochondrial permeability transition pore (mtPTP) [99].

H_2_O_2_ is less reactive than superoxide; despite this, it is potentially dangerous, being the substrate of the Fenton reaction, which produces the highly reactive hydroxyl radical. Therefore, other enzymes are involved in H_2_O_2_ removal, including catalase and glutathione, as well as thioredoxin peroxidase systems.

The enzyme catalase is found mainly within peroxisomes, and to a lesser extent, in mitochondria. Until now, it was described in heart [100] liver [101], and cerebral hippocampus [102]. Catalase breaks down two hydrogen peroxide molecules into one molecule of oxygen [103] and two molecules of water in a two-step reaction [104]. The first step involves the formation of an intermediate compound I (a covalent oxyferryl species (FeIVO)) with a porphyrin π-cation radical through the reduction of one hydrogen peroxide molecule [105]. In the second step, compound I is reduced through redox reactions by a two-electron transfer from the second molecule of hydrogen peroxide to produce the free enzyme, oxygen, and water [104].

Notwithstanding, catalase has the highest activity in decomposing the H_2_O_2_ in H_2_O and O_2_ and a low affinity for H_2_O_2_; therefore, it efficiently works when the inorganic hydroperoxide reach high levels [106].

It was found that the liver content of the catalase protein in the mitochondrial extracts increases with ageing, while its activity does not vary significantly. This result seems due to a significant increase in the rate of glycation that damage the enzyme, suggesting a link between glycation stress and the age-related decline in mitochondrial antioxidant defense [107].

In the cardiac mitochondria of mice, catalase contributes significantly to the consumption of H_2_O_2_ and is solely responsible for the removal of H_2_O_2_ in non-respiring or structurally damaged mitochondria [108]. Furthermore, in mice fed a high-fat diet, the mitochondrial catalase content increases by approximatively 50%, but this is not sufficient to prevent the H_2_O_2_-induced reduction in insulin signaling in the heart, revealed by reduced Akt phosphorylation stimulated by insulin. Thus, the selective increase in catalase does not prevent H_2_O_2_-induced loss in cardiac insulin signaling, indicating that mitochondrial catalase likely works to preclude the formation of high levels of H_2_O_2_ without perturbing redox-dependent signaling [109].

The enzymes, glutathione peroxidase (GPX) and peroxiredoxin (Prx), metabolize most of the H_2_O_2_. Their activities depend on the thiol groups of the residues of cysteine of reduced glutathione (GSH), and thioredoxin (Trx), respectively, to reduce the H_2_O_2_.

The GPXs comprise several phylogenetically related enzymes. Eight isoforms have been identified so far: the mammalian GPXs from 1 to 4 are seleno-proteins containing a selenocysteine in the catalytic center, GPX 6 is a seleno-protein only in humans and is expressed in the olfactory epithelium. GPX 5 contains cysteine instead of selenocysteine in the active center and is a secreted protein in the epididymis. GPX7 and GPX8 are cysteine-GPXs with a low GPX activity [110].

The selenol (-SeH) in seleno GPXs reacts as a selenolate with H_2_O_2_ to selenenic acid (-SeOH) that reduces back to -SeH by 2 GSH forming GSSG and water [111,112,113]. Among GPX, only GPX1 and GPX4 are found in mitochondria.

GPX1, a ubiquitously expressed homo-tetramer, is present in the cytosol and mitochondria. GPX1 is highly expressed in the mitochondria and the cytosol of the liver and kidney but poorly in the heart and muscle [114]. It works in a similar way to catalase by breaking down H_2_O_2_, but slowly and with a high affinity. Furthermore, it uses glutathione (GSH) as a reducing agent, which is converted into oxidized glutathione (GSSG) [115]. GPX1 reacts with hydrogen peroxide and low-molecular-mass, soluble hydroperoxides, but not with more complex lipid hydroperoxides [116] and decomposes the small quantities of peroxide that are produced in a continuous and physiological way inside the cells.

The genetic inactivation of GPX1 in mice casues a reduction of 20% in body weight compared to normal animals and increases the level of lipid peroxides in the liver. Moreover, liver mitochondria show an increased release of hydrogen peroxide and decreased mitochondrial respiratory control ratio and power output index. Hence, the genetic inactivation of GPX1 results in growth retardation, presumably due in part to reduced mitochondrial energy production as a product of increased oxidative stress [114].

GPX4 is a monomer able to reduce hydroperoxides in complex lipids, such as those inserted into bio membranes or lipoproteins [116]. There are three different isoforms of the enzyme: cytosolic, ubiquitous, mitochondrial (mGPx4), and sperm nuclear (snGPx4), expressed mainly in testis with only marginal amounts in other tissues [116].

GPx4 is synthesized in two forms, one long (23 kDa) and one short (20 kDa). The long form is localized in the mitochondria because it possesses a mitochondrial signal peptide, while the short GPx4 is localized in other cell organelles [117].

Whole GPx4 knockout mice do not survive, while mGPx4-only knockout mice are fully viable and develop normally, even though male mice are completely sterile [118].

GSSG produced by GPX activity cannot leave the mitochondria [119]. It is recycled back to GSH by the enzyme glutathione reductase (GR), which uses reduced nicotinamide adenine dinucleotide phosphate (NADPH) as a hydrogen donor [120]. GR contains two highly conserved domains: one binds FAD and NADPH, and the other is an interface dimerization domain. It also has two cysteines in the catalytic site, which form a disulfide bond. As expected, glutathione reductase tends to accumulate in cell regions of high electron flux, where reactive species are generated [120]. In eukaryotes it is found in the cytoplasm and within organelles including the nucleus and the mitochondria [121,122,123]. Glutathione reductase is translocated across the mitochondrial membranes from the cytosol. In human cells, a single gene expresses both the cytosolic and mitochondrial forms of glutathione reductase [124]. The GR destined for different cellular compartments is synthesized using alternative in-frame start codons; if the synthesis starts at the first AUG codon, a long form is obtained, and this isoform is marked for transport to the mitochondria. If translation starts at the second AUG codon, then the protein will remain in the cytosol [124].

The system of the thioredoxin comprises thioredoxin reductase (TrxR), thioredoxin (Trx), and peroxiredoxin (Prx). TrxR passes electrons from NADPH to Trx, whose reduced form donates an electron to Prx that, in turn, reduces hydrogen peroxide to water [125].

TrxRs are high-molecular-weight selenoenzymes [126]. Three iso-forms of TrxRs exist: the cytosolic TrxR1, the mitochondrial TrxR2 and the testicular TrxR3 or thioredoxin glutathione reductase [127]. The mitochondrial TrxR2 contains selenocysteine, is the key regulator of the thioredoxin system, and is essential for the redox homeostasis of the mitochondria [126,128,129].

The Trxs contain a thiol motif of the preserved active site. There are two isoforms, of which Trx1 is found mainly in the cytoplasm and nucleus and Trx2 in the mitochondria [130]. Both Trx2 and mitochondrial GSH/GSSG are redox systems distinct from the Trx1 and GSH/GSSG cytosolic systems [131,132].

Prxs are a large family of thiol-dependent peroxidases which, in the mammalian cell, comprise six isoforms [133]. Prx1, 2 and 6 are found in the cytoplasm, Prx4 in the endoplasmic reticulum, Prx3 in the mitochondria and Prx5 in various compartments, including peroxisomes and mitochondria [134].

The thioredoxin system is the primary defense against the peroxides produced in mitochondria, such as hydrogen peroxide and peroxynitrite [135]. TrxR2 role is to maintain Trx2 in its reduced state by using electrons from NADPH. Trx2 in turn is a cofactor of Prx3 and Prx5. Prx3 and Prx5 reduce H_2_O_2_ and peroxynitrite generated by mitochondrial metabolism [126,128,135,136,137,138].

In vascular endothelial cells, TrxR2 plays a key role in the control of vascular integrity. Its targeted loss causes disruption of both nitric oxide and redox homeostasis in vivo. When TrxR2 activity is impaired, the steady-state levels of peroxynitrite increase. This increase reflects the decreased bioavailability of NO· due to its O_2_^·−^-mediated oxidative inactivation. Thus, TrxR2 regulates peroxynitrite levels by providing reducing equivalents to mitochondrial peroxiredoxins which, in turn, catalytically reduce peroxynitrite to nitrite [139].

Cox and co-workers reported that approximately 90% of the H_2_O_2_ produced by mitochondria is metabolized by Prx3, and only a small but significant amount by Prx5 [140]. GPX1 reaction with H_2_O_2_ shows a rate constant higher and a lower mitochondrial concentration than Prx3. The low concentration restricts its power to compete directly with Prx3.

The GPX/GR and Trx/TrxR/Prx systems require NADPH as a source of electrons necessary for their activity. Several sources of NADPH exist. The first step of the pentose phosphate pathway, catalyzed by glucose-6-phosphate dehydrogenase (G6PD), was believed to be the primary source of NADPH generation. Other equally important enzymes weree described. These enzymes include the NADP+-specific forms of isocitrate dehydrogenase, malic enzyme, aldehyde dehydrogenase, and NAD kinase [141]. Furthermore, a direct trans-hydrogenation between NAD and NADP is verified in mitochondria [142].

The pool of NADPH and GSH in mitochondria is sufficient to counteract a sudden increase in ROS. However, the antioxidant defense system needs a continuous replenishment of NADPH and GSH, which is dependent on the capacity of the enzymes to restore these molecules.

#### 4.2.2. Low-Molecular-Weight Antioxidants

Mitochondria contain an efficient low-molecular-weight antioxidant system composed of molecules endogenously made or introduced with food.

##### Mitochondrial Glutathione

The tripeptide γ-glutamyl-cysteine-glycine (GSH) is the main ubiquitous non-enzymatic regulator of intracellular redox homeostasis. It synthetizes in the cytosol in a two-step reaction that requires metabolic energy. In the first reaction, the enzyme γ-glutamylcysteine synthetase catalyzes the reaction between glutamate and cysteine that leads to the formation of γ-glutamylcysteine. The first step is rate-limiting due to the generally low availability of cysteine and is also a regulatory step because GSH inhibits it. The GSH inhibition is necessary to maintain a proper GSH concentration, intracellularly [143,144]. In the second step, the enzyme GSH synthetase (GS) catalyzes the reaction between γ-glutamylcysteine and glycine. Part of the tripeptide synthesized in the cytosol transfers to cellular organelles such as the endoplasmic reticulum, nucleus, and mitochondria constituting separate redox pools, distinct from the cytoplasmic ones [145].

GSH can easily pass through the external mitochondrial membrane using porine channels. However, due to its anionic nature at physiological pH, GSH cannot diffuse through the inner mitochondrial membrane into the matrix due to the negative membrane potential of the intermembrane space. Consequently, the GSH of the mitochondrial matrix derives from the cytosol through a system located in the membrane that transports the GSH into the mitochondrial matrix against an electrochemical gradient. This agrees with the observation that the cytoplasmic GSH content decreases in some conditions, but the mitochondrial GSH content remains constant [146]. Two carriers, the dicarboxylate (DCc) and the 2-oxiglutarate (OGc), which exchange GS- with other anions so that no charges transport occurs through the membrane [147], were involved in GSH transport through the inner mitochondrial membrane in liver and kidney, to date [148,149]. In the liver, the transport of GSH mediated by OGc decreases in mitochondria from alcohol-fed rats and in liver mitochondria enriched in cholesterol [150]. These data suggest that OGc is sensitive to membrane dynamics [150]. However, other putative mitochondrial GSH carriers are still unknown [151]. Moreover, S-D-lactoylglutathione, an intermediate of the glyoxalase system, can enter the mitochondria and be hydrolyzed by the mitochondrial enzyme glyoxalase II to D-lactate and GSH. Thus, S-D-lactoylglutathione can represent an alternative source of mitochondrial GSH [152].

Mitochondrial GSH (mGSH) is only 10–15% of the cellular GSH but, due to the low volume of the matrix, its concentration (10 mM) is higher than the cytosolic GSH [146].

The reduction of the mitochondrial oxidized glutathione occurs due to the GR located in the matrix that uses as a reducing equivalent source the NADPH produced through NADP+ trans-hydrogenation, which is NADH- and energy-dependent [153]. In the presence of an electrochemical proton gradient, under physiological conditions, the reaction is strongly shifted towards NADPH formation, and the reaction rate is enhanced by 5–10 times [154].

Marì reviewed the contribution of mitochondrial GSH to disease progression in pathologies as diverse as Alzheimer’s disease, alcoholic and non-alcoholic steatohepatitis, and diabetic nephropathy [151].

##### Mitochondrial Coenzyme Q

Coenzyme Q or ubiquinone is a lipophilic molecule present in all tissues and cells, mainly located in the inner mitochondrial membrane. It is composed of a redox-active benzoquinone ring conjugated to an isoprenoid chain. The chain length differs among species. In humans, most ubiquinone contains 10 isoprenyl units and is designated CoQ10 [154]. Each cell synthesises its own CoQ. Isoprene units derive from the mevalonate pathway of acetyl CoA and assemble in polyprenylpyrophosphate [155]. The precursor of the quinoid head group is p-hydroxybenzoic acid derived from tyrosine or phenylalanine [155]. The head group of p-hydroxybenzoic acid conjugates to polyprenylpyrophosphate. Then, it produces the final CoQ molecule through decarboxylation, hydroxylation, and methylation steps [156,157]. The half-life of CoQ in vivo is about 50–125 h, and its breakdown product is a phosphorylated short-chain derivative, which is excreted in the urine [156,158]. How CoQ is mobilized from its site of synthesis on the inner mitochondrial membrane to other action sites remains a longstanding mystery. Recently, two highly conserved but poorly characterized mitochondrial proteins, Ypl109c (Cqd1) and Ylr253w (Cqd2), which affected this process in yeast, were identified [159].

CoQ carries electrons from complex I and II to complex III of the mitochondrial respiratory chain. It also functions as a fat-soluble antioxidant, scavenging reactive oxygen species and is involved in multiple aspects of cellular metabolism [160]. Indeed, the reduced form of coenzyme Q (ubiquinol) also acts as an effective antioxidant in biological membranes, as suggested by the observation that ubiquinol and α-tocopherol lower the lipid peroxidation in mitochondrial suspensions to the same extent [161].

The antioxidant properties of CoQ10 also depend on its capacity in recycling other antioxidants such as vitamin C and vitamin E [162]. CoQ10 deficiency is found in chronic and age-related diseases. For instance, in cardiovascular diseases (CVDs), the reduced bioavailability of CoQ10 is because statins, one of the most common lipid-lowering drugs, inhibits the common pathway shared by CoQ10 endogenous biosynthesis and cholesterol biosynthesis [162].

CoQ also functions as a cofactor for the H^+^ transport by the uncoupling proteins (UCP-1, 2 and 3) [163,164] that dissipate chemiosmotic gradients as heat. The uncoupling, in turn, reduces the reduction levels of electron carriers, lowering ROS production.

Finally, CoQ influences the opening of the permeability transition pore (PTP) a mitochondrial channel whose opening causes the collapse of the mitochondrial membrane potential (ΔΨ) leading to apoptosis. Physiological and biochemical studies showed that the effects of cardio protection and the prevention of the apoptosis of CoQ10 may also be due to the direct inhibition of the opening of the mitochondrial PTP during the reperfusion of ischemic heart [165,166]. Indeed, animals’ treatment with CoQ10 decreases disturbances of heart function and normalizes oxygen metabolism after ischemia reperfusion. This effect is accompanied by a substantial stabilization of the mitochondrial membrane. Moreover, the incubation of mitochondria, isolated from rat heart, with CoQ10 (0.01 μM) substantially prevents calcium- and oxidant-induced mitochondrial swelling [165].

Furthermore, CoQ10 treatment reduces apoptosis induced by apoptotic stimuli. This reduction is accompanied by the inhibition of mitochondrial depolarization, the release of cytochrome c and the activation of caspase 9, events caused by the opening of the mitochondrial PTP suggesting that the antiapoptotic activity of CoQ10 could be related to its ability to prevent this phenomenon [166].

#### 4.2.3. Diet-Derived Mitochondrial Antioxidants

The mitochondrial antioxidant defenses also comprise molecules deriving from the diet. Such substances can reach and accumulate in mitochondria.

Vitamin C (Vit C) is a water-soluble vitamin. It is also named ascorbic acid (AA) or ascorbate. Vit C is produced in plant cells but synthesized endogenously in animal species, except for humans, monkeys, bats, guinea pigs, and some reptiles [167]. Humans lost this capability because of a series of inactivating mutations of the gene encoding gulonolactone oxidase, a key enzyme for the biosynthesis of Vit C [168]. Animals for which ascorbic acid is a vitamin acquire it from food sources via a substrate-saturable transport mechanism. The oral Vit C intake determines plasma concentrations that are tightly regulated. With Vit C oral intake exceeding 200 mg daily, the plasma vitamin C concentration does not increase further through increasing the oral intake [169]. The maximal plasma concentration induced by the oral intake of Vit C is about 200 µM. In healthy humans, the physiological plasma concentrations of the vitamin ranges from 40 to 100 µM [169,170]. Although the plasma level of vitamin C is in the micromolar range under physiological conditions and with the typical dietary intake, the intracellular level of the vitamin is in the millimolar range. This high concentration is due to the selective intracellular accumulation through a transport system of vitamin C present in the plasma membrane [171].

Vit C concentration in mammalian mitochondria increases in dietary vitamin C supplementation [172,173,174]. This increase depends on the presence of specialized mitochondrial mechanisms of uptake. The carrier of the oxidized form of the vitamin, dehydroascorbic acid (DHA), was initially identified as the facilitative glucose transporter 1 (GLUT1) [175]. Moreover, GLUT10, another glucose transporter, which is expressed highly in the mitochondria of smooth muscle cells and insulin-stimulated adipocytes facilitates the transport of DHA. DHA can protect against oxidative stress. This protection is compromised when GLUT10 expression in mitochondria is inhibited [176].

In a subsequent study, a mitochondrial ascorbic acid transporter (MAT) from both rat liver and potato mitochondria was reconstituted in proteoliposomes, showing that this was not a GLUT since it showed different biochemical features [177]. The protein has a molecular mass in the range of 28–35 kDa, and catalyzes the saturable, temperature, pH-dependent, unidirectional transport of both ascorbic acid (AA) and DHA. The transport activity is sodium-independent, and it is optimal at acidic pH values. It is stimulated by the proton gradient, thus supporting the idea that ascorbate is symported with H^+^ [177].

The dehydroascorbic acid (DHA) that enters into the mitochondria is reduced and accumulated as mitochondrial AA (mtAA). Mitochondrial ascorbate can deactivate ROS, and thus protect both the mitochondrial genome and membranes from oxidative damage [175]. Ascorbate is recycled in mitochondria by various mechanisms, including the reduction by α-lipoic acid and/or thioredoxin reductase, or a GSH-dependent reduction. The latter is one of the main mechanisms of ascorbate recycling [178].

Vitamin E is the main lipid-soluble antioxidant in the cells. It comprises tocopherols and tocotrienols that contain a chromanol ring with a 13-carbon chain at the C2 position. Vitamin E synthesizes in plants as four homologues differing for the number and localization of methyl groups attached to the chromanol ring (named α, β, γ, and δ). Tocopherols have a saturated side chain and three chiral carbons, resulting in eight stereoisomers. Tocotrienols have an unsaturated side chain with three double bonds and one chiral carbon forming two stereoisomers [179,180].

Vitamin E absorbed in the intestine enters the circulation through the lymphatic system. It is packaged together with the lipids in chylomicrons. At this stage, there are no differences in the plasma levels of the different forms of vitamin E. It enters the hepatocytes by endocytosis of the remnants of chylomicron and reaches the late endosomal compartment. Preferentially, the α-tocopherol (α-T) binds to an α-tocopherol transfer protein (αTTP), that localizes on the outer leaflet of the endosomal membrane. The bond between vitamin E and αTTP favors the transfer of α-tocopherol to the plasma membrane. Here, the binding to the resident phosphatidylinositol 4,5-bisphosphate determines a conformational change, resulting in the release of α-T and its incorporation into the membrane [181,182,183]. Subsequently, vitamin E bonds with the ATP-binding cassette transporter 1 (ABCA1) that allows vitamin E to exit the cells, be inserted into lipoproteins, and delivered to extrahepatic tissues [181]. α-TTP has a large affinity for α-tocopherol (100%) and a much lower affinity for β-, γ- and δ-tocopherol (50%, 10–30%, or 1%, respectively) [184]. A large portion of non-α-T forms of vitamin E is oxidized to quinones or conjugated with glucuronic acid and excreted in the feces [185]. The hydrolysis of VLDL by lipoprotein lipase delivers α-tocopherol to extrahepatic tissues and yields low-density lipoproteins (LDLs). LDLs, which carry the major portion of plasma α-tocopherol, provide a further route for the delivery of α-tocopherol to extrahepatic tissues via the LDL receptor-mediated uptake pathway [186]. Vitamin E also localizes in the membrane of organelles, such as the endoplasmic reticulum, mitochondria, and peroxisomes [187,188,189,190,191].

The supplementation with vitamin E increases its content in mitochondria from the liver, skeletal and cardiac muscle [192,193,194]. It was proposed that vitamin E reaches the mitochondrial membrane through passive diffusion, which seems to be the main driving force for the distribution of vitamin E within the cell [195].

Vitamin E exerts antioxidant effects in different ways. It can deactivate oxygen singlet (^1^O_2_) by quenching, and one molecule of α-tocopherol can deactivate up to 120 ^1^O_2_ before its degradation [196]. Vitamin E is a potent, chain-breaking antioxidant that chemically scavenges ^1^O_2_ and lipid peroxyl radicals. In the first case, it irreversibly produces quinones and epoxides. In the second case, it converts in the tocopheroxyl radical [197], which can be recycled back to α-tocopherol by ascorbate. In this way, tocopherols trap propagating radical intermediates produced during lipid peroxidation and break the chain reactions of radicals.

The protective effects of vitamin E from oxidative damage also depend on its capacity to scavenge superoxide radicals, thus downregulating mitochondrial ROS production [198]. The α-tocopheryl radical produced in such a reaction seems to be repaired by a superoxide radical [199]. Following α-tocopherol-administration, the O_2_^•−^ release rate of mouse submitochondrial particles from the liver and skeletal muscle is inversely related to the α-tocopherol content [200]. The rate of H_2_O_2_ released by mitochondria isolated from liver and skeletal muscle is reduced following vitamin E supplementation in a dose-dependent manner [201]. Moreover, in intact mitochondria, the reduction in the H_2_O_2_ release rate is associated with the lowering in both indexes of oxidative damage to lipids and proteins and the susceptibility to in vitro oxidative stress [192]. In membranes, a ratio of one tocopherol per thousand of polyunsaturated fatty acid side chains is normal; therefore, the vitamin E protective effects are obtained with a low vitamin membrane concentration [202]. The mitochondrial antioxidant power may also depend on other fat-soluble antioxidants that can localize in the mitochondria these include astaxanthin, a red pigment that belongs to the subclass of xanthophylls, which are able to counteract mitochondrial dysfunction as they are able to permeate and co-localize in the mitochondria [203]. It was shown that astaxanthin prevents mitochondrial dysfunction due to oxidative stress and mitigates oxidative stress in various pathological conditions [203].

#### 4.2.4. Mitochondrial Systems of Repair

The accumulation of oxidatively damaged macromolecules in mitochondria is prevented by an efficient system deputed to their repair.

Lipids of the mitochondrial membranes are continuously exposed to ROS and are highly susceptible to oxidative damage.

Most mitochondrial lipids are synthesized in the endoplasmic reticulum (ER) and transported to the mitochondria, but cardiolipin and phosphatidylethanolamine are synthesized within the inner membrane of the mitochondria and are critical for maintaining the architecture of the mitochondrial cristae [204].

The glycerophospholipid of the inner mitochondrial membrane, cardiolipin, acts as an anchor for respiratory supercomplexes and mitochondrial DNA during replication. Cardiolipin is essential for mitochondrial health [205]; its oxidation is reported as a primary event in the release of cytochrome c and the increase in the permeability of the mitochondrial membrane to apoptosis factors [206]. Moreover, when lipid oxidation is associated with iron overload, ferroptosis takes place, which is a form of iron-dependent cell death [207].

Cardiolipin peroxidation and peroxidated lipid degradation products reduce the activities of the respiratory chain complexes. Moreover, they promote the opening of the mitochondrial transition pore and mitochondrial permeability transition [208]. GPX4 is the key enzyme involved in the protection of mitochondrial lipids from the effects of peroxidation. Its overexpression counteracts the lowering of the inner membrane potential and ATP production in conditions that lead to oxidative stress [208]. More recently, another mechanism that can contribute to containing mitochondrial lipid oxidation was suggested. This mechanism involves the ubiquinol-mediated repair of mitochondrial peroxidated lipids [209]. The enzyme, dihydroorotate dehydrogenase, catalyzes the conversion of dihydroorotate to orotate in a reaction of oxidation, in which ubiquinone is converted into ubiquinol. Therefore, indirectly, dihydroorotate dehydrogenase can protect cells from lipid peroxidation by generating ubiquinol, which acts in the repair of oxidative damage to mitochondrial lipids [209].

Oxidatively modified proteins can accumulate in mitochondria at high levels both in basal and stress conditions. The proteins are oxidatively modified in an irreversible way, i.e., proteins containing carbonyl groups must be eliminated to prevent the genesis of insoluble aggregates, which can be dangerous for the mitochondria. There are a number of mitochondrial systems that can identify and remove oxidatively damaged polypeptides.

Twenty or more proteases constitute the mitochondrial proteolytic system, which is involved in several functions [210]. The control of the quality of the mitochondrial proteins is the main function of the mitochondrial proteolytic system that allows the extension of the half-life of mitochondria. Some proteases, localized both in the intermembrane space and in the matrix, play different roles. They regulate the ratios of subunits of mitochondrial complexes that are encoded by nuclear and mitochondrial DNA; eliminate damaged, unfolded, or misfolded proteins; and control the protein turnover [211].

Two complexes of proteases act in the quality control of protein across the inner mitochondrial membrane, called membrane-bound AAAs complexes (ATPases associated with a wide variety of cellular activities) [212]. These membrane-embedded peptidases are named m- and i-AAA proteases for their different topologies in the inner membrane; the m-AAA protease is active in the matrix and the i-AAA protease on the intermembrane side of the membrane [212]. Other peptidases contribute to the quality control of the inner membrane, one of which is the metallopeptidase OMA1 [213]. An ATP serine protease, Lon protease, degrades denatured or oxidatively damaged proteins in the matrix [214,215], avoiding oxidized proteins that accumulate in the mitochondria of all human tissues, particularly in the heart, brain, liver, and skeletal muscles. The age-dependent decline in the activity and regulation of this proteolytic system may underlie the accumulation of oxidatively modified and dysfunctional proteins and loss in mitochondrial viability [216].

Several kinds of chemical modifications can damage DNA. These modifications include the spontaneous deamination and base loss, non-enzymatic alkylation and enzymatic methylation, adducts formation with aromatic molecules, intra- and inter-strand cross-links, protein–DNA adduct formation, and oxidation [217,218].

Mitochondria possess their own DNA genome (mtDNA) that encodes only for 13 polypeptides, which are essential components of four of the five complexes of the respiratory chain. Each mitochondrion contains between 2 and 10 molecules of DNA, which are organized as nucleoids [219]. ROS can produce a variety of DNA damages such as oxidized DNA bases, abasic sites, and double-strand breaks (DSBs). The damages in the mitochondrial DNA can have harmful effects, including mitochondrial diseases, ageing and age-related diseases. The replication of damaged mtDNA can lead to cellular damage. Therefore, mitochondria adapted mechanisms to repair damaged DNA. These mechanisms rely on nuclear-encoded DNA repair proteins that are translocated into the mitochondria [220]. The repair of mtDNA damage relies on Base Excision Repair (BER), Homologous Recombination (HR) and Microhomology-mediated End Joining (MMEJ). These repair mechanisms do not operate in isolation, and evidence for the interplay between pathways exist [221].

BER is initiated by a DNA glycosylase that recognizes and cleaves the glycosidic bond between damaged nitrogenous bases and the pentose moiety. Then, the site is cleaved by endonuclease lyase in order to cleave the DNA backbone. Following the DNA polymerase and DNA ligase, the repair is completed. Mitochondrial BER enzymes are encoded by the nuclear DNA, mostly existing as splice variants or as proteins with post-translational modifications [222].

While nuclear BER decreases with age, the mitochondrial BER may increase with age. This increase is not sufficient to prevent the gradual accumulation of lesions in the mitochondrial DNA with age.

HR and MMEJ play a central role in the double-strand breaks (DSBs) repair in mitochondria [223,224].

## 5. The Nuclear Factor Erythroid 2–Related Factor 2 (Nrf2) and Mitochondrial Antioxidants

Nuclear factor erythroid 2–related factor 2 (Nrf2) regulates cytoprotective responses to stress induced by electrophilic compounds and ROS [225].

In cells not exposed to a stress condition, the Nrf2 protein levels are low because proteasomes degrade it after ubiquitination. The Kelch-like, ECH-associated protein 1 (Keap1) is the main negative regulator of Nrf2 and mediates the ubiquitination and degradation of Nrf2 [226,227]. In conditions of stress, or the presence of Nrf2-activating compounds, the cysteine residues of Keap1 oxidize and Keap1 loses its ubiquitin ligase activity. This modification allows for the release of Nrf2 that is phosphorylated and moves into the nucleus. Here, Nrf2 binds to small proteins of musculoaponeurotic fibrosarcoma (Maf) and forms heterodimers that bind to the antioxidant response element (ARE) in the promoter regions of Nrf2-regulated genes [228,229].

Another model of the Nrf2-Keap1 pathway proposes that Nrf2 is primarily a nuclear protein and that it is expressed and constitutively recruited into chromatin to drive basal gene expression. Keap1 appears to repress Nrf2 activity by transiently moving into the nucleus to promote its ubiquitination. The steady-state level of Nrf2 is maintained by a dynamic pathway that balances its constitutive expression with a degradation process regulated by Keap1 downstream of its role as a transcriptional activator [230]. Under stressful conditions, Keap1 does not pass into the nucleus where Nrf2 accumulates and increases transcription. This model explains how Nrf2 exerts its dual function of controlling gene expression in a constitutive and inducible way.

Whatever the gene regulation pathway operated by Nrf2, this certainly is the main factor regulating antioxidant defenses. Indeed, it regulates more than 200 cytoprotective genes in response to oxidative stress [231].

Nrf2 activation induces mitochondrial antioxidant enzymes such as thioredoxin reductase-2 (TrxR2), peroxiredoxin 3 (Prx3) and 5 (Prx5), and SOD2 [232].

In some circumstances, the activation of mitochondrial antioxidant gene expression by Nrf2 requires other partners to verify or is secondary to the Nrf2-induced expression of other nuclear factors. In mice with Staphylococcus aureus-induced peritonitis, the co-activator peroxisome proliferator-activated receptor-gamma 1α (PGC-1α) acts as a co-activator for Nrf2 during Sod2 induction [233]. In this condition, PGC-1α plays a key role in regulating hepatic mitochondrial biogenesis and in protecting against mitochondrial oxidative stress.

Moreover, in vitro studies using trabecular meshwork cells demonstrated that the quercetin induction of Prx3 and Prx5 expression was verified through the Nrf2 induction of nuclear respiratory factor 1 (NRF-1) [234].

Nrf2 can also affect mitochondrial characteristics. Nrf2 induces mitochondrial biogenesis; mitophagy increases mitochondrial antioxidant capacity and activates oxidative phosphorylation regulating substrate availability [232,235,236,237].

Nrf2 regulates some key metabolic genes or crosstalk with other transcription factors. Nrf2 activation enhances glycolytic flux, pentose phosphate pathway, amino acid metabolism, and glutaminolysis. These Nrf2-induced activations determine an increased flux of substrates and reduce equivalents into the Krebs’s cycle and the mitochondrial respiratory chain. The genetic activation of Nrf2 in cells increases the oxygen consumption rates, mitochondrial membrane potential (∆Ψm), ATP levels (Holmstrom et al., 2013) and fatty acid oxidation [238]. Nrf2 upregulates the uncoupling protein 3 (UCP3), which is associated with decreased superoxide formation [239]. Nrf2 activation can also protect mitochondrial DNA (mtDNA), as shown in intestinal epithelial cells subjected to ischemia reperfusion-induced damage [240].

Experimental evidence suggests that Nrf2, in addition to antioxidant responses, is involved in many other cellular processes, including metabolism and inflammation, and its functions are beyond those originally envisioned [241].

Nrf2 involvement in mitochondrial biogenesis is verified through the activation of NRF-1, which induces the transcription of the mitochondrial transcription factor A (TFAM) [242]. NRF-1 activates the transcription of several mitochondrial genes, in particular genes encoding the subunits of the mitochondrial respiratory chain complexes and the transcription of TFAM, which regulate the replication and transcription of the mitochondrial genome [243].

Murata et al. [244] showed that Nrf2 regulates the expression of the PTEN-induced putative kinase 1 (PINK1), an essential factor for controlling mitochondrial quality and protecting cells from oxidative stresses. Damaged mitochondria arising from stress conditions induce the Nrf2-dependent transcription of the PINK1 gene through the production of ROS. PINK1 accumulates on the outer membrane of depolarized mitochondria. Accumulated PINK1 recruits Parkin, a ubiquitin ligase, and Parkin can mediate the autophagic elimination of depolarized mitochondria [245]. PINK1 is necessary for mitochondrial quality control and contributes to mitophagy [246,247].

Some data indicate that mitochondrial ROS (mtROS) can activate Nrf2 in some conditions and deactivate it in others. Therefore, it was suggested that a high level of mtROS inactivates the signalling cascade that activates Nrf2, thus leading to the inhibition of Nrf2 activity [248]. Furthermore, a role in Nrf2 inhibition can also be played by ROS that are produced in the matrix or intermembrane space or even by the type of ROS produced. Further studies are needed to clarify these aspects [248].

## 6. Mitochondria Can Remove ROS Produced by Other Cellular Sources

The mitochondrial antioxidant system seems to remove not only the ROS produced by mitochondria, but also the O_2_^•−^ and H_2_O_2_ produced in the surrounding medium [5].

The ability of mitochondria to remove H_2_O_2_ depends on respiration and is different in the mitochondria of different tissues. Moreover, it changes depending on the metabolic state and the respiratory substrate. Zoccarato et al., showed that brain mitochondria remove exogenous H_2_O_2_ faster with malate and glutamate than with succinate as substrates of the respiration [249]. Moreover, they found that H_2_O_2_ removal in the State 3 of respiration was slightly lower than in State 4. The H_2_O_2_ removal supported by succinate is nearly the same in State 4 and in State 3 of respiration. They suggested that the H_2_O_2_-removing capacity of respiring mitochondria depends primarily on the activities of GPX and GR, and that other antioxidant systems only contribute ~20% of detoxifying activity [249].

Later, this idea was questioned by Dreshel and Patel, who found that the glutathione system makes only a minimal contribution to the brain’s mitochondrial removal capacity. Using pharmacological inhibition of mitochondrial antioxidant enzymes, they found that the rate of H_2_O_2_ removal only decreased by 25% after GR inhibition, and GPX inhibition had no effect [250]. Conversely, the inhibition of TrxR caused a reduction in the H_2_O_2_ removal rates by 80%, and the oxidation of peroxiredoxin lowered this rate by 50%. Non-enzymatic mitochondrial processes also contributed to the removal of hydrogen peroxide but to a lesser extent (estimated to be about 10%) [250].

Other studies showed that also liver mitochondria removed H_2_O_2_ and that such removal was verified at higher rates in the presence of respiratory substrates. However, the H_2_O_2_ removal rates are similar with succinate or pyruvate/malate, substrates linked to complex II and I, respectively [251] (Venditti et al., 2014). The selective pharmacological inhibition of antioxidant enzymes shows that catalase is the major contributor to H_2_O_2_ removal (~31%) and that TrxR and GPX also contribute significantly, but to a lesser extent (~20% and 23%, respectively) [252]. The contribution of non-enzymatic processes to H_2_O_2_ removal is about 27% and appears to depend mainly on haemoproteins, as suggested by its changes in the mitochondria of animals with different cytochrome c content [252]. Indeed, it was shown that, in hypothyroid and hyperthyroid conditions, the H_2_O_2_ removal rate from liver mitochondria is lower and higher, respectively, compared to the euthyroid ones. These changes were associated with similar changes in cytochrome c content and in the contribution of non-enzymatic systems to peroxide removal [252].

During state 4 of respiration, the mitochondria of the heart and liver remove H_2_O_2_ at similar rates [253]. In cardiac mitochondria, enzymatic antioxidant systems contribute to the removal of H_2_O_2_ to the same extent as hepatic mitochondria, but, unlike the liver, the non-enzymatic system contributes to a greater extent (~36.8%). This effect is consistent with the higher content of hemoproteins [253].

## 7. Mitochondrial ROS Removal and ROS Signaling

So far, it is established that ROS can regulate various cell signaling pathways and numerous physiological processes [254]. This suggests that maintaining the adequate cellular levels of H_2_O_2_ is of prime importance for cell function and survival.

The different characteristics of mitochondria render them capable of controlling the cytosolic level of H_2_O_2_ [255]. First, mitochondria can maintain high levels of NADPH in the matrix, which is necessary for the activities of most of the H_2_O_2_-metabolizing enzymes. To the maintaining of such high level a main contribution is due to the respiration-dependent transhydrogenase. Secondly, the resistance of the Prx3 and 5 located in the matrix to oxidative inactivation, unlike cytosolic Prx1, makes the mitochondria capable of removing H_2_O_2_ even if H_2_O_2_ levels are high. Third, mitochondria possess high amounts of mitochondrial GSH and antioxidant enzymes. For these reasons, it was hypothesized that mitochondria possess several redox mechanisms that allow them to potentially play an important role in the modulation of H_2_O_2_-activated signals in the cytosol, facilitating their desensitization [247].

## 8. Conclusions

Until recently, mitochondria were regarded only for their role as producers of aerobic energy in eukaryotic cells. Indeed, they are the sites of oxidative phosphorylation, the process by which the transfer of electrons from energy substrates to oxygen is coupled with the synthesis of ATP. Later, the observation that the flow of electrons in the respiratory chain also determines the genesis of ROS capable of causing damage to cellular components gave rise to the idea that mitochondria are involved in the degenerative phenomena that cause various diseases and ageing.

Subsequently, another role emerged for the mitochondria, as a system capable of protecting the cell from oxidative damage. Indeed, several data suggest that the mitochondrial systems evolved to protect mitochondria from the ROS they produce can also eliminate ROS produced by other cellular sources. It can be hypothesized that this action, which is particularly important in physio-pathological conditions, in which the cellular production of ROS occurs to a greater extent, is more effective in tissues that have an abundant mitochondrial population. Moreover, several experimental evidence, suggested that mitochondria can control the cellular H_2_O_2_ levels which make them regulators of the ROS-mediated signaling pathways.
